# Surgical treatment of a pleomorphic adenoma in the hard palate of a young female adult: a case report

**DOI:** 10.1097/RC9.0000000000001194

**Published:** 2026-10-22

**Authors:** Yousra Zemmouri, Soukaina Bahbah, Riad Hariri, Saliha Chbicheb

**Affiliations:** Department of Oral Surgery, Faculty of Dental Medicine, Mohammed V Universityin Rabat, Rabat, Morocco

**Keywords:** case report, hard palate, minor salivary glands, oral surgery, pleomorphic adenoma

## Abstract

**Introduction:**

Pleomorphic adenoma (PA) is the most common benign salivary gland tumor, and it rarely involves the minor salivary glands of the hard palate.

**Methods:**

This case report has been reported in accordance with the Surgical CAse REport (SCARE) 2025 guidelines.

**Case presentation:**

A 21-year-old woman presented with a painless, slowly growing palatal mass. Clinical and radiological findings suggested a benign lesion. Wide local excision was performed, and histopathology confirmedPA.

**Clinical discussion:**

Wide local excision is essential to prevent recurrence. Healing by secondary intention provided complete re-epithelialization without functional impairment.

**Conclusion:**

Adequate surgical management ensures excellent outcomes.

## Introduction

Pleomorphic adenoma (PA) is the most common salivary gland tumor, accounting for approximately two-thirds of all salivary gland neoplasms^[^1^]^. PA accounts for approximately 40–70% of all salivary gland tumors and 60–65% of benign salivary gland neoplasms^[^2^]^. Minor salivary gland involvement represents only 10–25% of all PA, with the hard palate being the most common intraoral site. Occurrence in young adults is relatively uncommon, making such cases particularly noteworthy^[^3^]^.

Also known as a benign mixed tumor due to its epithelial and myoepithelial components, PA predominantly affects the major salivary glands, particularly the parotid gland. Among tumors arising from minor salivary glands, the palate is the most frequently involved site, followed by the lips, buccal mucosa, gingiva, and tongue^[^4,5^]^.

HIGHLIGHTS

- Pleomorphic adenoma of the hard palate is a rare tumor of the minor salivary glands.
- Clinical presentation is typically a slow-growing, painless palatal mass.
- Wide local excision is essential to prevent recurrence.
- Secondary-intention healing can achieve excellent functional outcomes.
- Long-term follow-up is necessary to detect recurrence.

Clinically, PA usually presents as a slow-growing, painless, and well-circumscribed mass covered by normal mucosa. Ulceration is uncommon and generally related to local trauma. Unlike PAs of major salivary glands, those arising from minor salivary glands often lack a true capsule, which increases the risk of incomplete excision and recurrence. Wide local excision, including the periosteum and underlying bone when necessary, is therefore considered the treatment of choice. Although benign, PA carries a risk of malignant transformation estimated at approximately 6%^[^6^]^. Early diagnosis and appropriate surgical management are essential to prevent recurrence and malignant progression.

This case is of particular interest because of the young age of the patient, the palatal location requiring preservation of phonetic and stomatognathic functions, and the favorable outcome following surgical management.

## Case presentation

A 21-year-old patient presented to the Oral Surgery Department of a tertiary care hospital for evaluation of a palatal swelling that had been progressively increasing in size over the preceding months. The patient reported no pain, functional discomfort, or episodes of bleeding.

Intraoral examination revealed a well-circumscribed, firm, non-tender mass located on the right posterolateral hard palate, measuring approximately 2 cm in diameter. The overlying mucosa appeared intact, with a slight violaceous discoloration (Fig. 1). No ulceration or surface changes were observed. A preoperative panoramic radiograph was obtained and showed no evidence of underlying maxillary bone destruction, tooth displacement, or other associated osseous abnormalities (Fig. 1).

**Figure 1.**
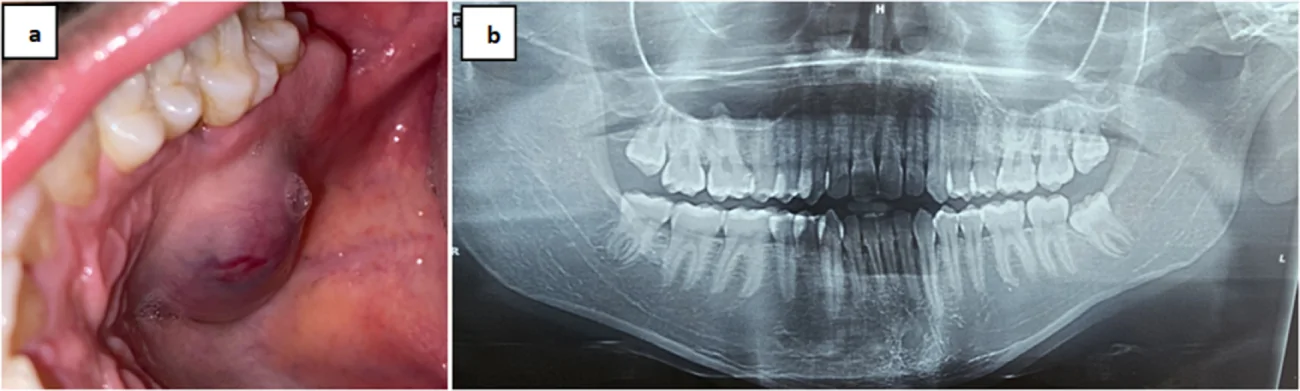
(A) Preoperative intraoral photograph showing a palatal mass on the right hard palate. (B) Preoperative panoramic radiograph demonstrating the absence of underlying bone involvement.

An aspiration test was carried out during the initial consultation and yielded no cystic or purulent content. The clinical and radiographic findings suggested a benign salivary gland tumor.

Surgical excision was undertaken under local anesthesia. A mucoperiosteal flap was elevated, exposing the lesion and revealing an underlying bone depression consistent with pressure-induced bone resorption. The mass was carefully dissected and removed in its entirety along with its pseudocapsule. The excised specimen, a firm and lobulated mass measuring approximately 2 × 1.5 cm, was submitted for histopathological evaluation (Fig. 2).

**Figure 2.**
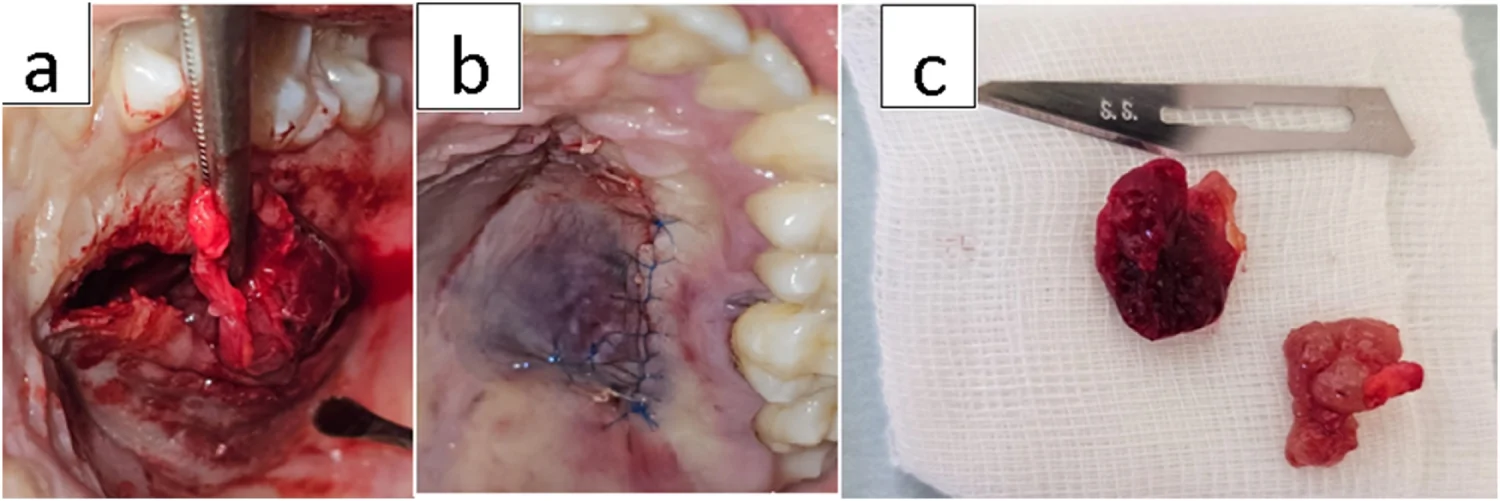
(A) Intraoperative view showing exposure of the palatal lesion and the underlying bone depression following flap elevation. (B) Immediate postoperative appearance of the surgical site after complete excision of the tumor and suturing. (C) Gross specimen of the excised pleomorphic adenoma, demonstrating a firm, lobulated mass measuring approximately 2 × 1.5 cm.

Microscopic examination demonstrated a biphasic epithelial and myoepithelial proliferation embedded in a myxoid and focally chondroid stroma, confirming the diagnosis of PA. No cytological atypia, necrosis, or significant mitotic activity were observed. Given the characteristic histopathological features, the diagnosis was established based on conventional histopathological examination, and immunohistochemical analysis was not performed. Surgical margins were free of tumor (Fig. 3).

**Figure 3.**
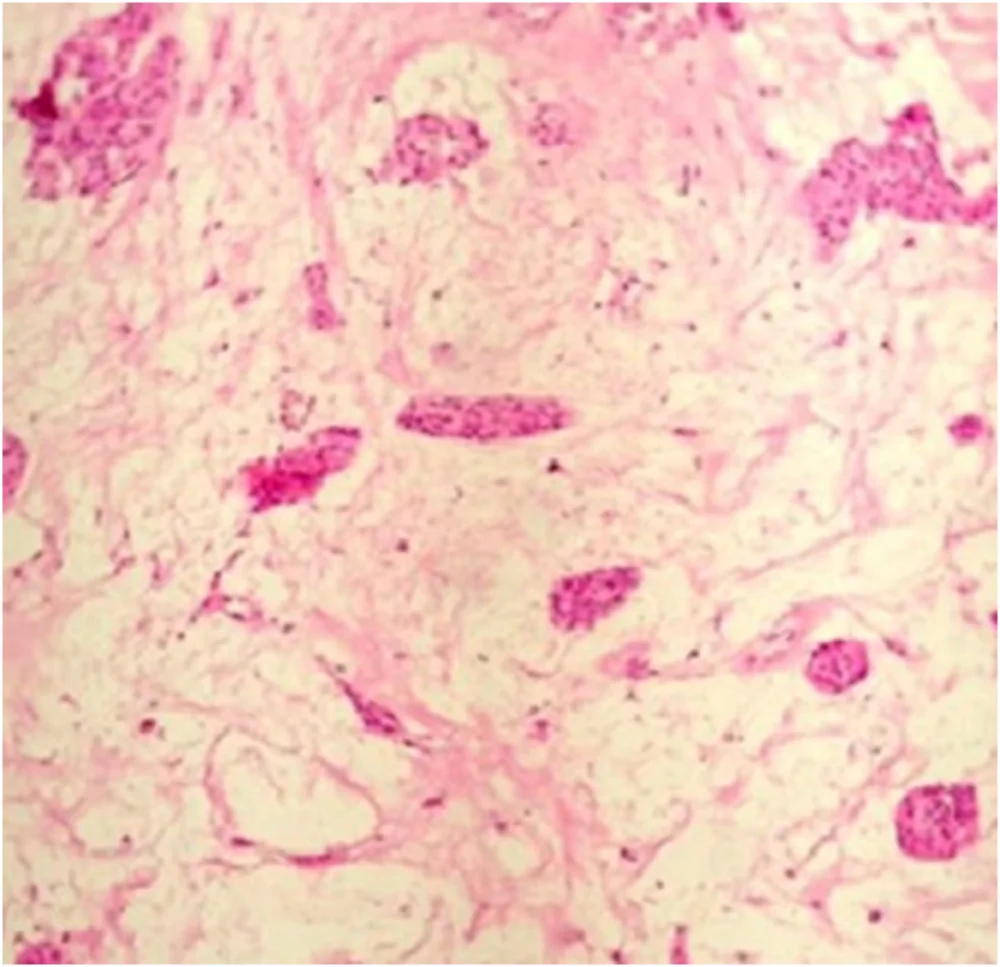
Microphotograph showing epithelial and myoepithelial proliferation within a myxoid stroma, characteristic of pleomorphic adenoma. H&E, ×40.

Postoperative healing was uneventful, with complete mucosal healing achieved 2 months after surgery. Differential diagnoses included palatal abscess, mucocele, and other benign salivary gland tumors.

## Discussion

The prevalence of salivary gland tumors ranges from 20% to 40%^[^7^]^. Tumors arising from the minor salivary glands are more likely to be malignant. The most affected age group is between 40 and 60 years, with a higher prevalence in females. This condition is most commonly observed in the hard and soft palates due to the high concentration of minor salivary glands in this region^[^8^]^.

Clinically, PA typically presents as a firm, unilateral, asymptomatic mass with slow growth, which may become large if left untreated. When it develops in the minor salivary glands, it is most frequently located in the hard and soft palate, again reflecting the high density of salivary glands in this area. It usually appears as a firm or elastic submucosal mass, without ulceration of the overlying mucosa^[^6^]^.

Histopathological analysis is essential to confirm the diagnosis of PA. The classic PA consists of a mixture of epithelial, myoepithelial, and stromal components within a myxoid, chondroid, or fibrous stroma. The epithelial component often forms ductal and cystic structures or may appear as cellular islands or sheets^[^9^]^.

The treatment of choice for PA is wide local excision, including resection of the periosteum or bone when involved. Simple enucleation is not recommended due to the high risk of recurrence, which is related to the possible persistence of microscopic tumor extensions beyond the apparent capsule^[^10^]^. Palatal reconstruction is considered in cases of extensive palatal defects resulting from the surgical excision of highly aggressive tumors.

In our case, complete excision of the tumor was performed, resulting in a palatal defect. The postoperative course was marked by delayed healing; however, tissue repair progressed gradually by secondary intention, with satisfactory re-epithelialization and without fistula formation or functional complications^[^11^]^. Recurrence of these tumors is rare when adequate surgical excision is achieved^[^12^]^. However, recurrences may occur after enucleation, as the absence of a true capsule can lead to the persistence of microscopic pseudopod-like extensions. Spiro reported a recurrence rate of 6% in his study involving 1342 patients with benign tumors of the minor salivary glands^[^13^]^.

Although PA is a benign tumor, malignant transformation has been reported in approximately 5–6% of long-standing or recurrent cases, highlighting the importance of complete surgical excision and long-term follow-up.

The uniqueness of the present case lies in the patient’s young age, as PA of the palate is more commonly reported in middle-aged adults. Furthermore, complete healing was achieved following surgical excision, with no evidence of recurrence during follow-up.

## Conclusion

PA of the hard palate arising from the minor salivary glands is an uncommon entity that requires accurate diagnosis and appropriate surgical management. Wide local excision remains the cornerstone of treatment to prevent recurrence. This case demonstrates that, even in the presence of delayed healing, secondary-intention healing can provide excellent functional and esthetic outcomes without the need for complex reconstructive procedures. Careful long-term follow-up is essential to detect possible recurrence and ensure sustained clinical success.

## Data Availability

Data sharing is not applicable to this article as no datasets were generated or analyzed.
